# Analysis of gene repair tracts from Cas9/gRNA double-stranded breaks in the human CFTR gene

**DOI:** 10.1038/srep32230

**Published:** 2016-08-25

**Authors:** Jennifer A. Hollywood, Ciaran M. Lee, Martina F. Scallan, Patrick T. Harrison

**Affiliations:** 1 Department of Physiology, University College Cork, Cork, Ireland; 2 Department of Microbiology, University College Cork, Cork, Ireland

## Abstract

To maximise the efficiency of template-dependent gene editing, most studies describe programmable and/or RNA-guided endonucleases that make a double-stranded break at, or close to, the target sequence to be modified. The rationale for this design strategy is that most gene repair tracts will be very short. Here, we describe a CRISPR Cas9/gRNA selection-free strategy which uses deep sequencing to characterise repair tracts from a donor plasmid containing seven nucleotide differences across a 216 bp target region in the human CFTR gene. We found that 90% of the template-dependent repair tracts were >100 bp in length with equal numbers of uni-directional and bi-directional repair tracts. The occurrence of long repair tracts suggests that a single gRNA could be used with variants of the same template to create or correct specific mutations within a 200 bp range, the size of ~80% of human exons. The selection-free strategy used here also allowed detection of non-homologous end joining events in many of the homology-directed repair tracts. This indicates a need to modify the donor, possibly by silent changes in the PAM sequence, to prevent creation of a second double-stranded break in an allele that has already been correctly edited by homology-directed repair.

The foundations of contemporary gene editing were laid with two key observations from a plasmid-based study of DNA homologous recombination-dependent DNA repair pathways. The first was the finding that two exogenous DNA molecules containing non-overlapping deletion mutants of the bacterial aminoglycoside 3′-phosphorylase (Neo^R^) gene could recombine in mammalian cells such that one plasmid served as a template or donor for the precise repair or editing of the other. The second was that the creation of a double-stranded break (DSB) close to the genetic lesion in the Neo^R^ gene in one of the plasmids using a restriction enzyme prior to transfection considerably enhanced the frequency of homologous recombination^1^.

Whilst proof-of-principle that an exogenous DNA molecule could be used as a template to precisely edit an endogenous genomic sequence was soon established by successful deletion of exon 8 of the hypoxanthine phosphoribosyl transferase gene in mouse embryo-derived stem cells^2^, experiments to determine the effects of a DSB were more difficult to test due to the lack of endonucleases with sufficient specificity to cut at a unique genomic location. To address this, Jasin and colleagues engineered a human cell line with a Neo^R^ gene containing both a premature stop codon and a unique 18 bp recognition site for the I-*Sce*I meganuclease^3^. Upon co-transfection of an I-*Sce*I expression vector to create a targeted DSB, and a donor plasmid to act as a template to correct the mutant Neo^R^ gene, they found that gene editing as measured by the production of neomycin-resistant clones, was stimulated by three orders of magnitude compared to cells transfected with the donor alone^4^. By using donor plasmids with up to eight single nucleotide changes, each creating a novel restriction site without changing the coding sequence of the Neo^R^ gene across a 745 bp region, they were also able to map the extent of repair tract length; analysis of 80 recombinant clones revealed that the majority (75%) of repair tracts were 12 bp or shorter^4^. Although they also reported a small number of very long tracts in the same study (up to 511 bp), this seminal analysis of repair tracts, along with the original observation from plasmid studies that a DSB close to the target site enhances recombination frequency, have become widely cited in gene editing studies as the rationale for creating DSBs at or close to the target sequence to be modified when using a donor sequence for homology-directed repair (HDR) with programmable and/or RNA-guided endonucleases^5^,^6^,^7^,^8^,^9^.

We have previously reported^10^ the use of template-dependent zinc finger nuclease (ZFN) gene editing to correct the most common cystic fibrosis-causing mutation, F508del, a 3 bp in-frame deletion in the CFTR gene^11^. Although we observed a high level of ZFN-induced DSBs, the level of editing at the F508del site was at least an order of magnitude lower, which we speculated was most likely a consequence of the large distance (203 bp) between the ZFN target site and the F508del mutation.

Subsequent studies of template-dependent editing of the F508del mutation have utilised powerful selection strategies to successfully correct this mutation in both human gut stem cells^12^ and human iPS cells^13^,^14^,^15^. However, since these enrichment strategies are not compatible with direct *in vivo* application of gene editing^16^, we decided to further characterise template-dependent editing without using a selection approach. Here, we describe a CRISPR Cas9/gRNA strategy to allow the correction of the F508del mutation and characterisation of repair tracts either side of the Cas9-induced DSB by deep sequencing. We observed that 90% of the template-dependent repair tracts were >100 bp with equal numbers of uni-directional and bi-directional repair tracts. Use of a selection-free system also allowed us to detect and characterise template-independent non-homologous end joining (NHEJ) events that occur both independently, and in combination with HDR events.

## Results

### gRNA design and expression

**Figure 1:**
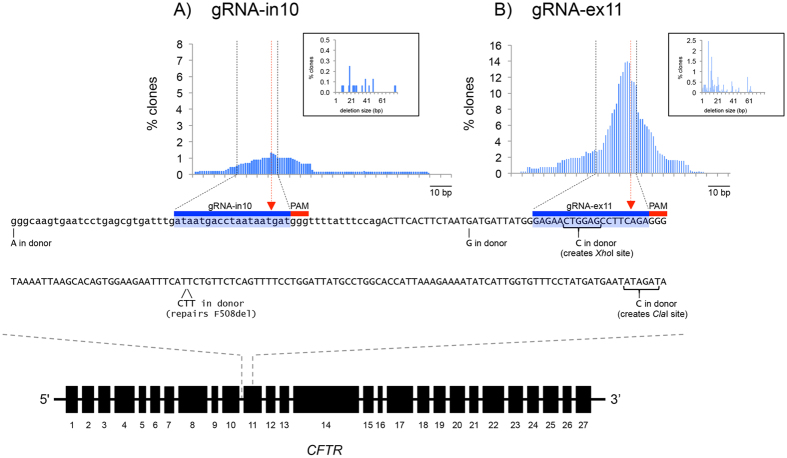
NHEJ activity in target region of CFTR. The lower panel shows the location of the gRNA target sites within a 216 bp sequence of the CFTR gene spanning intron 10 (lowercase)/exon 11 (UPPERCASE) boundary. The PAM motif for each gRNA target is overscored in red, and the 19 bases bound by the gRNA are overscored in blue. The red triangles indicate the predicted DSB location. Below the sequence are the seven nucleotides which are different from this sequence in the donor plasmid; they include two SNPs, two single base changes to create *Xho*I and *Cla*I restriction sites, and the three base-pair CTT sequence to repair the F508del mutation. The upper panel is a graphical representation of the incidence of deletions caused by NHEJ in CFTE cells following expression of Cas9 and either (**A**) gRNA-in10 or (**B**) gRNA-ex11. The graphs plot the deletions at each nucleotide position as vertical lines expressed as the percentage of reads carrying deletions as quantified by next generation sequencing. Black dashed lines demarcate boundaries of the gRNA targeting sites and red dashed lines indicates predicted DSB site. Insets in A and B are graphical representation of the frequency of deletion size. Mean (±SEM) deletion size for gRNAin10 was 32 ± 4 bp (median = 25). Mean (±SEM) deletion size for gRNA-ex11 was 19 ± 1 bp (median = 13).

To analyse the repair tracts of CRISPR/Cas9 gRNA-induced double-stranded breaks (DSBs), we developed a gene targeting assay designed to repair the F508del mutation in human trachael epithelial cells (CFTEs) derived from a cystic fibrosis patient homozygous for this mutation^17^. We designed two CRISPR guide RNA (gRNA) target sequences that match the GN_20_GG consensus sequence^18^,^19^ in a ~200 bp region of the CFTR gene that spans the intron 10/exon 11 boundary and includes the 3 bp in-frame CTT deletion site which causes the F508del mutation (Fig. 1). To clone and express the gRNAs, we created a one-step cloning vector (see Supplementary Figure S1), based on the U6 promoter-target RNA-guide RNA scaffold plasmid described by Mali and colleagues^19^, but modified to include two *Bse*RI sites to enable directional Golden Gate cloning^20^. As the last G residue in the U6 promoter is part of the 3′ overhang generated by the first of the two *Bse*RI sites, DNA fragments made from two oligodeoxynucleotides that encode gRNAs can be cloned without the need to include an additional G at the 5′ end.

### Cas9/gRNA cleavage and repair by non-homologous end joining (NHEJ)

To measure the ability of the CRISPR/Cas9 system to create DSB in the CFTR gene, CFTE cells were co-transfected with plasmids encoding Cas9 and either gRNA-in10 or gRNA-ex11. Seventy-two hours post-transfection genomic DNA was analysed by deep sequencing to determine the frequency and size of deletions caused by NHEJ repair of Cas9/gRNA-induced DSBs. As shown in Fig. 1A, the gRNA that targets intron 10 of *CFTR* resulted in deletions in 1.3% (21/1609) of alleles. In contrast, the gRNA that targets exon 11 of *CFTR* resulted in a higher deletion rate of 14.3% (192 out of 1346 alleles; Fig. 1B and Supplementary Figure S2). In both cases the deletions are centred at a site approximately 3 bp upstream of the PAM (consistent with the predicted Cas9 cut site), and the majority (70%) of deletions range in size from 4 to 24 bp (Fig. 1 insets). These levels of NHEJ repair and deletion size distribution are similar to that reported in other epithelial cell lines such as 293 cells^19^. No deletions were observed in deep sequencing analysis of mock transfected cells.

### Cas9/gRNAex10 template-dependent editing and repair tract analysis

To evaluate template-dependent editing and characterise the DNA repair tracts, we used a donor plasmid that contains a 216 bp sequence of CFTR centred around the gRNAex11-induced DSB, which includes seven nucleotide differences from the genomic target sequence in CFTE cells in this region (see Fig. 1), and is flanked by ~2 kb homology arms^10^. The donor plasmid was co-transfected into CFTE cells with Cas9 and gRNAex11 plasmids, and 72 h later, genomic DNA was extracted and analysed by deep sequencing.

**Figure 2:**
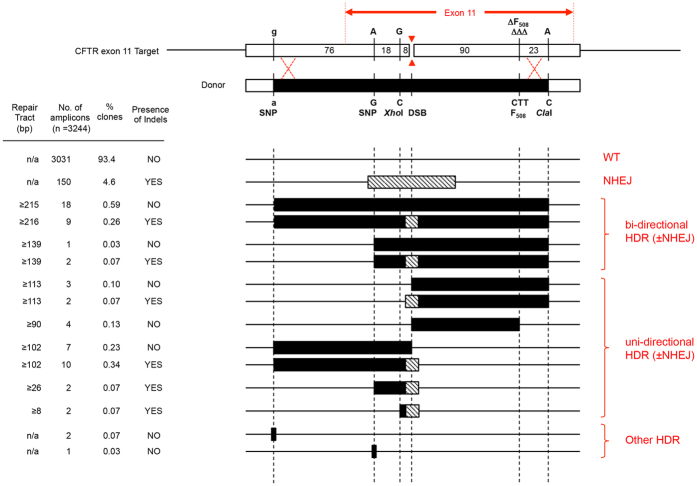
Schematic representation of DNA repair tracts. The upper panel is a representation of the 216 bp target region of the CFTR gene shown in Fig. 1 which lacks the 3 bp CTT sequence. Underneath is a representation of the 219 bp region of the donor plasmid which shows the approximate position of the seven nucleotides which differ from the target sequence, including the CTT which should repair the F508del mutation. The red arrows indicate the position of the DSB created by the RuvC (top arrow) and HNH (bottom arrow) domains of Cas9. The lower part of the figure shows the different repair tracts observed, tract length, observed frequency and presence or absence of Indels.

As shown in Fig. 2, 1.9% (63 out of 3244) amplicon sequences showed evidence of donor-dependent editing, a similar efficiency to that reported with two different Cas9/gRNA combinations to correct mutations in a GFP reporter gene^19^. Detailed analysis of the 63 gene-edited tracts by deep sequencing showed two predominant categories of repair. The first comprised 30 long continuous bi-directional tracts, the vast majority of which extend >100 bp either side of the DSB created by Cas9/gRNAex11 as shown by the incorporation of the SNPs and restriction enzyme sites from the donor; in all these bi-directional repair tracts, the 3 bp deletion which causes the F508del mutation in the CFTR protein, located 87 bp downstream of the DSB, was repaired. The second group comprised 30 unidirectional repair tracts, 87% of which were ≥90 bp in length. Nine of these unidirectional tracts occurred in a downstream direction and extend ≥90 bp from the DSB (as evidenced by repair of the F508del mutation which inserts the 3 bp sequence CTT), whereas the other 21 unidirectional repair tracts occurred in an upstream direction; of these, 17 were also long (≥102 bp). A third and minor category (5% of total) was identified comprising three upstream non-continuous repair tracts, two of which had only the G residue in the target sequence replaced by A from the template 102 bp upstream of the DSB, and one with the A residue in the target sequence replaced by G from the template 26 bp upstream of the DSB; none of these tracts had the G to C editing event to create an *Xho*I site located 8 bp upstream of the DSB.

The use of deep sequencing also allowed us to analyse repair of DSBs by non-homologous end joining. In addition to the 63 amplicon sequences which showed evidence of homology-directed repair (HDR), a further 150 amplicons showed evidence of NHEJ. Closer examination of the 63 template-dependent editing events revealed that just under half also showed evidence of NHEJ events ranging from a 16 bp deletion to a 1 bp insertion (see Fig. 2 and Supplementary Figure S3). If the NHEJ and HDR events that occur in a single amplicon are considered as separate editing events, then the overall ratio of NHEJ:HDR events is 2.8, comparable to the 2.9 ratio reported using the essentially the same CRISPR/Cas9 gRNA vector system to repair human iPS cells with single-stranded oligodeoxynucleotide donors^21^.

## Discussion

Using the human CFTR F508del mutation as a model system, we performed template-dependent editing with *S. pyogenes* Cas9 and a gRNA that creates a DSB 87 bp upstream of the 3 bp CTT deletion (F508del mutation) and a donor plasmid designed to analyse gene repair tracts of ~100 bp both before and after the DSB. We analysed these tracts without enrichment or selection for editing events by deep sequencing of the target genomic region. The overall level of template-dependent editing was 1.9%, and 90% of these events were long continuous repair tracts in excess of 100 bp from the DSB with no bias towards bi-directional or uni-directional correction.

Our observations that a donor plasmid allows efficient gene editing even when the desired nucleotide change is >100 bp from the Cas9/gRNA cut site, means a wider selection of gRNAs are potentially available to target a particular sequence. It also means a single gRNA could be used with variants of the same template to create or correct specific mutations within a 200 bp range. For example, in the case of exon 11 of CFTR, which is 192 bp long, there are at least 10 other CF-causing mutations in the CFTR2 database^22^ which could be corrected using the same donor and gRNA combination described herein. Given that 80% of human exons are <200 bp long^23^, use of a single gRNA/donor combination could be useful to correct such a range of mutations in an individual exon, particularly if a gRNA can be identified in that region with a very low level of off-target binding^24^. It also means that in the unlikely event that a 200 bp stretch of DNA does not contain an *S. pyogenes* Cas9 PAM sequence (5′-NGG-3′) on either strand, it should still be possible to edit efficiently by using a PAM just outside of this region. Efficient template-directed gene editing at a distance would also increase the utility of Cas9 with PAMs that have longer and therefore less frequently occurring recognition sequences such as NNGRR(T) in *Staphylocccus aureus*^25^ and NNNNGATT in *Neisseria meningitidis*^26^. The corollary of these potential advantages of efficient gene editing at distances of ≥100 bp from the DSB is that unwanted changes may inadvertently be introduced into the genome if the homology arms are not identical to the target region, though this is easy to avoid by sequencing both alleles prior to homology arm design^27^.

**Figure 3:**
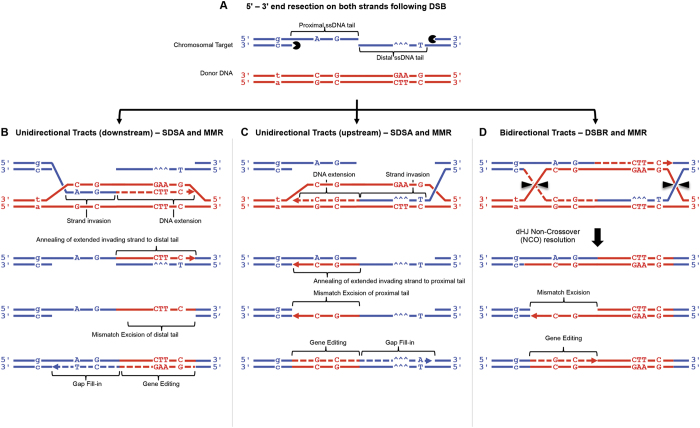
Mechanisms of gene editing. (**A**) Schematic representation of chromosomal target (blue), donor plasmid (red), SNPs and 3 bp deletion (^^^)/3 bp insertion (CTT). Target shown with 5′ ends resected and proximal/distal 3′ ssDNA tails exposed. Donor DNA is shown with the top strand in the 3′-5′ direction. (**B**) Mechanism of unidirectional tract repair occurring downstream of the DSB. The proximal ssDNA tail invades the donor DNA creating a D-loop, and the invading strand extends incorporating CTT and C SNP from the donor. Once sufficiently extended, the invading strand leaves donor and anneals to distal ssDNA in chromosome creating gapped duplex. Following mismatch removal (from same strand as the gap), DNA synthesis by pol δ would result, in this example, in a downstream editing tract containing both the CTT sequence and C SNP. (**C**) Mechanism of unidirectional tract repair occurring upstream of the DSB. The distal ssDNA tail invades the donor DNA creating a D-loop, and the invading strand extends incorporating G and C SNPs from the donor (reading in the 5′-3′ direction). Once sufficiently extended, the invading strand leaves the donor and anneals to the proximal ssDNA tail in chromosome creating a gapped duplex. Following mismatch removal (from same strand as the gap), DNA synthesis by pol δ would result, in this example, in an upstream editing tract conaining the G and C SNPs closest to the DSB are edited, whereas the g SNP in the intron is not edited. Longer or shorter tracts are explained by length of DNA extension prior to annealing with the ssDNA tails. (**D**) Mechanism of bidirectional tract repair spanning both sides of the Cas9 can be initiated by either of the ssDNA tails; this example starts with distal ssDNA tail invasion creating a D-loop, and incorporation of SNPs from the donor as before. Rather than collapse, the D-loop expands and captures the second strand as a template for DNA synthesis resulting in two Holliday junctions (HJs). Non-crossover resolution creates an edited downstream tract, with heteroduplex DNA in the upstream region processed by mismatch removal and DNA synthesis to complete the editing process.

A variety of pathways have been proposed to explain DNA repair of a nuclease-induced DSB, but the process of synthesis-dependent strand annealing (SDSA), first described in the study of P element-induced gap repair in Drosophila^28^, and also known as single end invasion^29^, appears to offer the best explanation of gene editing when the donor that has homology arms which perfectly match the target sequence on both sides of the DSB^8^,^30^. However, SDSA alone does not appear sufficient to explain the formation of uni-directional and bi-directional gene conversion tracts which arise when gene editing is performed with donors comprising homology arms with a low level (~1%) of heterology^4^,^31^; in this situation, mismatch repair (MMR) mechanisms are also proposed to play a role in resolving the heteroduplexes formed during recombination^32^,^33^. A combination of SDSA and MMR offers an explanation of both the upstream and downstream uni-directional repair tracts we describe here. The first step of the repair process is the resection of both 5′-ends at the DSB (Fig. 3A), creating proximal and distal 3′ single-stranded DNA (ssDNA) exposed tails^34^. The second step is single end invasion of the donor DNA by one of the exposed 3′ ssDNA tails which creates a displacement loop (D-loop)^32^,^33^. The invading DNA strand is then extended using the DNA sequence of the donor plasmid as a template. If the D-loop collapses before the 2^nd^ strand of the chromosome is captured, the newly synthesised DNA strand anneals to the ssDNA tail on the other side of the DSB. The creation of downstream editing tracts commences with strand invasion by the proximal 3′ ssDNA tail such that the newly synthesised DNA would extend by 90 bases before incorporating the CTT triplet (which corrects the CFTR F508del mutation) and then the C SNP of the *Cla*I site (Fig. 3B). Upon annealing to the proximal ssDNA tail, a heteroduplex DNA tract is created, which if then excised and filled-in by mismatch repair (MMR), would account for the unidirectional repair tracts that occur downstream of the DSB (Figs 2 and S3). Any strands which anneal but cease extension before incorporating the CTT sequence from the donor could potentially repair the DSB, but would not be detected by deep sequencing. The creation of upstream editing tracts starts with strand invasion by the distal 3′ ssDNA tail such that the newly synthesised DNA could be extended to include the C SNP of the *Xho*I site, then the G SNP, and ultimately the A SNP 102 bp upstream of the DSB (Fig. 3C). Thus, upon annealing to the proximal ssDNA tail, a heteroduplex DNA tract is created, which if then excised and filled-in by mismatch repair (MMR), would account for the unidirectional repair tracts that occur upstream of the DSB (Figs 2 and S3). In situations where the D-loop does not collapse, this would enable a second-strand capture event and formation of a double Holliday junction (dHJ) structure (Fig. 3D shows second capture derived following D-loop formed following single end invasion by distal 3′ ssDNA tail). Non-crossover resolution of this dHJ structure, followed by mismatch repair would account for the bi-directional repair tracts observed (Figs 2 and S3).

These models however do not explain the small number (3/63) of uni-directional template-dependent gene editing events on the 5′ side of the gRNA target site. These three repair tracts show incorporation of either the A residue from the template 102 bp upstream of the DSB or the G residue from the template 26 bp upstream of the DSB, but none of them contain G to C editing event located 8 bp upstream of the DSB. The discontinuous nature of these tracts argues against strand invasion and DNA synthesis from the DSB, however, they could be explained by donor-directed mismatch repair of a single stranded nick in the DNA^35^. Given that all three of these repair tracts occur upstream of the DSB, this suggests that the nick occurred on the complementary strand, which in CRISPR editing is defined as the DNA strand which has the complementary sequence to the gRNA, and would therefore be created by the HNH domain of Cas9^36^,^37^,^38^.

Our finding that the 90% of repair tracts are ≥100 bp differs from the finding in the seminal study of homology repair tracts^4^ which showed that the 75% of repair tracts are ≤12 bp. Indeed, this study has guided many nuclease-directed template-dependent gene editing strategies to create DSBs close to the target region to be repaired^5^,^6^,^7^,^8^,^9^. However, it should be noted that Elliott and colleagues also reported many examples of repair tracts in excess of 100 bp (in one case up to 511 bp), the majority of which were bi-directional. Although the factors which control repair tract length are not fully understood^39^, a number of differences between our approach and that of Elliott and colleagues^4^ may contribute to the divergence in the ratio of long:short repair tract length observed between the two studies. In addition to using Cas9/gRNA rather than I-*Sce*I, a key difference is that we used a strategy that monitors repair tracts without the need for selection, whereas Elliott and colleagues^4^ characterised repair tracts only in cloned cells in which a modified neomycin resistance gene which contained an I-*Sce*I target site with an in-frame stop codon had been successfully corrected. A second difference relates to the level of sequence identity between target and template at the site of the DSB. In our system, the first few bases of the invading 3′ ends created by the Cas9/gRNA DSB and subsequent end-resection have complete sequence identity to the donor template and would allow strand extension without the need for removal of 3′ nonhomologous sequences^40^,^41^. In contrast, the first few bases of the invading 3′ ends created at the I-*Sce*I site are non-homologous with the donor template (which contains an *Nco*I site at the equivalent location^4^), so these 3′ nonhomologous sequences (9 bases from proximal ssDNA tail, 13 bases from distal ssDNA tail) are most likely resected in order to allow DNA synthesis^40^,^41^. If this resection process extends just 8 bases beyond the region of non-homology into the distal tail (the location of the first distal tract marker), this would enable the generation of short homology tracts by SDSA alone (without the need for MMR), which could contribute to the higher ratio of short:long tracts observed in that study.

The overall level of correction of the F508del mutation in just 1.9% of amplicons is still relatively low. However, as transfection efficiency was roughly 30%, then the editing efficiency per transfected cells is expected to be ~3-fold higher; use of a single vector^7^ to express both gRNA and Cas9 may also improve editing efficiency. The high level of NHEJ events in HDR repair tracts also strongly suggests the need to modify the donor, possibly by silent changes in the PAM sequence, to prevent creation of a second DSB in an allele that has already been correctly edited by HDR. We chose not to modify the PAM sequence in the donor plasmid used here as any change to the NGG sequence would also have disrupted the glycine codon at position 480 of CFTR protein (See Supplementary Figure S2); use of Cas9 from other species with a longer PAM sequence could give more options for modifying the donor without affecting the coding sequence.

In summary, we have shown that in a selection-free system, Cas9/gRNA template-dependent editing gives rise to predominantly long continuous repair tracts with bi-directional and uni-directional events occurring with roughly equal frequency. This allows greater flexibility in selecting gRNAs within a ~200 bp window for template-dependent editing, and suggests that for 80% of exons in the human genome, a single gRNA could be used to create or correct all mutations within an exon.

## Materials and Methods

### Cell lines and Transfections

The cystic fibrosis tracheal epithelial (CFTE) cell line^17^ was obtained from Dieter Gruenert (UCSF) and maintained in modified Eagle’s medium (supplemented with 10% foetal calf serum, 1% l-glutamine, and 1% penicillin and streptomycin; Sigma) and incubated at 37 °C and 5% CO_2_. 300,000 cells were transfected with a total of 4 μg plasmid DNA and 10 μl of Lipofectamine 2000 (Invitrogen).

### Plasmids and Golden gate cloning

The donor plasmid was described previously^10^ and was transfected as intact circular supercoiled molecules. The pGUIDE gRNA expression vector was made by direct DNA synthesis of a 474 bp sequence (see Supplemental Figure S1) into the *Not*I sites of the pEX-A plasmid (Eurofins Genomics). dsDNA fragments encoding the target sequence of the gRNA (see Supplemental Figure S1) were assembled from two oligos and cloned into pGUIDE by Golden Gate assembly using *Bse*RI/T4 DNA ligase (NEB). Plasmid hCas9^19^ was a gift from George Church (Addgene plasmid #41815).

### DNA Repair Tract Detection and Quantification by Deep Sequencing

Genomic DNA was isolated using DNeasy DNA extraction kit (Qiagen). A 2.6-kb PCR product was generated using Platinum pfx (Invitrogen) and primers FP-i9.1 and RP-i10.1. A second PCR was performed using sequencing labelled CFTR-NHEJ^For^ and CFTR-NHEJ^Rev^ primers to produce a 432 bp product which was subjected to next generation sequencing analysis.

Genomic DNA was isolated and treated with *Dpn*I (NEB) for 90 min at 37 °C to restrict plasmid DNA. A 2.6-kb PCR product was generated using Platinum Taq HF (Invitrogen) and primers 5′-AATTTTGTAAATTTGTTTCATC-3′ and 5′-ACTTGCTTTGCCATTAACAGA-3′. 435-bp amplicons for sequencing by GS FLX++ chemistry (Eurofins Genomics), were generated with the primers 5′-ATCATGTGCCCCTTCTCTGT-3′ and 5′-CGTAGACTAGTGCTTTGATGACGCTTCTGTAT-3′ tagged with a unique 10 nucleotide multiplex-identifier (MID). Sequence alignments were performed using Clustal W^42^ and MegAlign (Version 11.2.1. DNASTAR. Madison, Wisconsin).

## Additional Information

**How to cite this article**: Hollywood, J. A. *et al*. Analysis of gene repair tracts from Cas9/gRNA double-stranded breaks in the human CFTR gene. *Sci. Rep.* **6**, 32230; doi: 10.1038/srep32230 (2016).

## Supplementary Material

Supplementary Information
