# Supplementary material for: Analysis of gene repair tracts from Cas9/gRNA double-stranded breaks in the human CFTR gene
Source: Sci Rep. 2016 Aug 25;6:32230. doi: 10.1038/srep32230 (PMC4997560; doi:10.1038/srep32230)
Supplement: Supplementary Information [file srep32230-s1.pdf]

Analysis of gene repair tracts from Cas9/gRNA double-stranded breaks in the human CFTR gene

Jennifer A Hollywood, Ciaran M Lee, Martina F Scallan and Patrick T Harrison

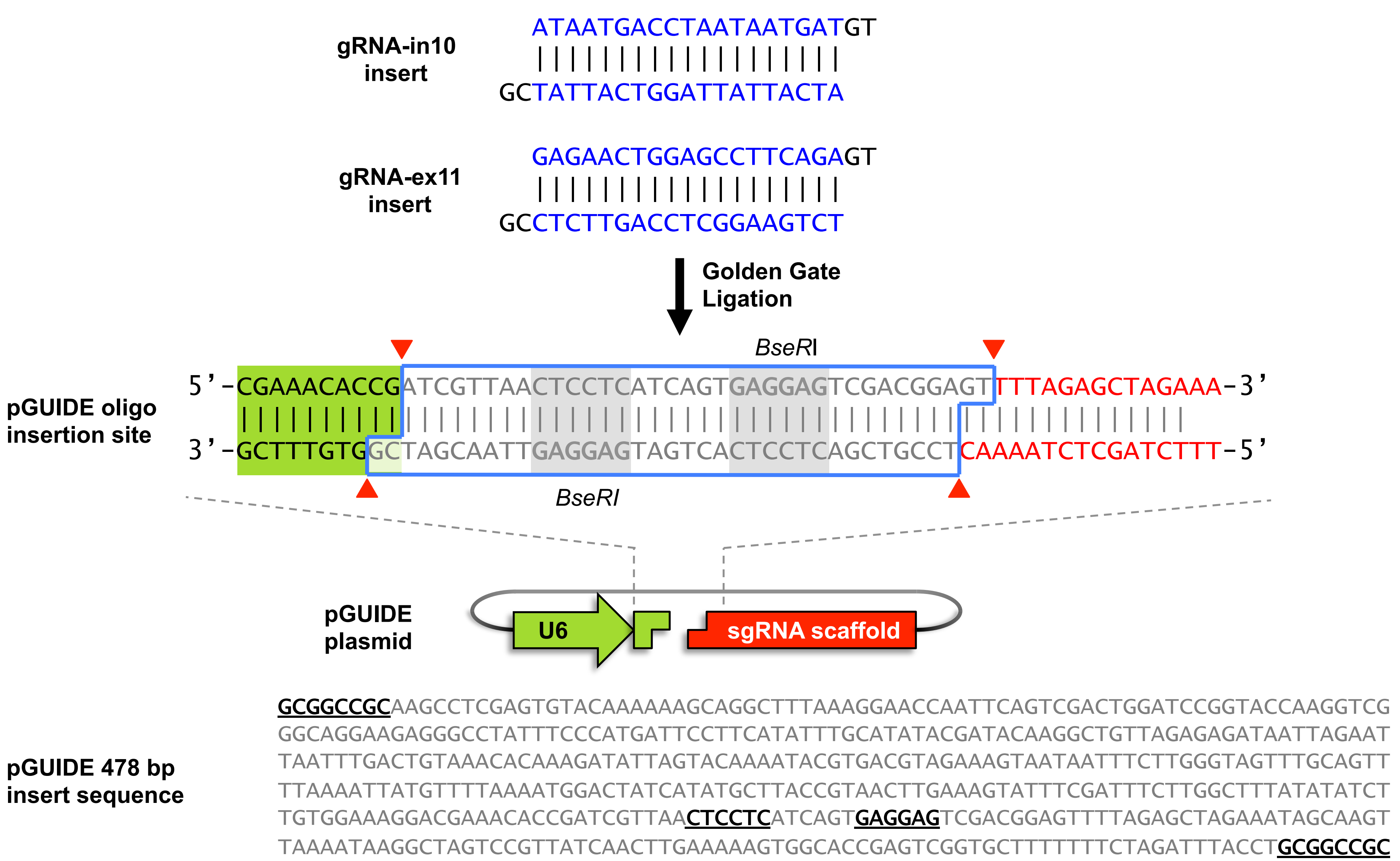

Figure S1. Oligonucleotide pairs used to encode gRNAs, and schematic representation of cloning into plasmid which contains sgRNA scaffold (pGUIDE). The upper oligonucleotide sequence in blue in each pair correspondes to the 19 bp sequence preceding 5′-NGG in genomic DNA. Sequences in black represents overhangs for ligation into the pair of *Bse*RI sites in pGUIDE. Golden gate cloning into the *Bse*RI sites enables direct insertion of annealed oligos in the correct orientation with the U6 promoter and ensures a G-C basepair for efficient U6 transcription. Lower panel shows the 478bp DNA sequence of the insert with the *Not*I sites and *Bse*RI sites underlined.

Figure S2. Sequence alignments of 101 bp region of all indel-containing amplicons from Cas9/gRNA-ex11-treated cells arranged in order of (a) increasing deletion size and (b) decreasing insertion size. The wild-type (WT) DNA sequence and corresponding amino acid sequence of this region is shown at the top of each list, PAM sequence in red, gRNA target region in blue, insertions highlighted in green. Nucleotides in intron 10 are shown in lowercase, and nucleotides in exon 11 are shown in uppercase. Note that the two essential guanosine residues of the *S. pyogenes* Cas9 NGG PAM sequence are the first two nucleotides of the glycine codon at position 480 in CFTR.

a) List of gRNA-ex11 deletions

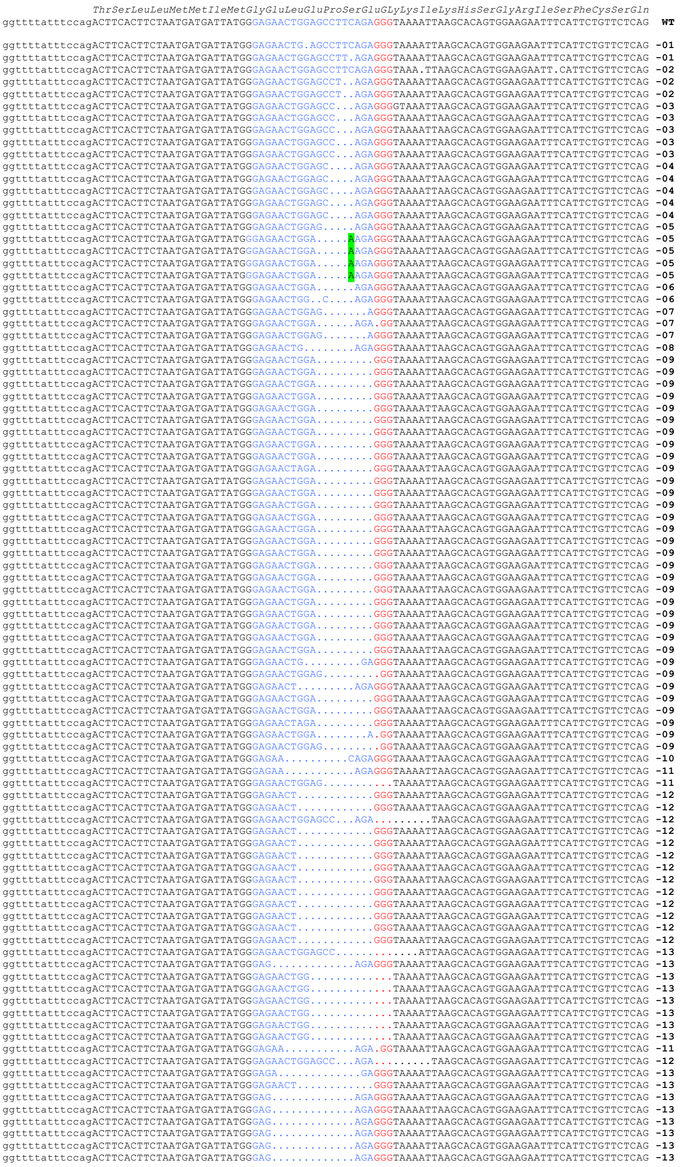

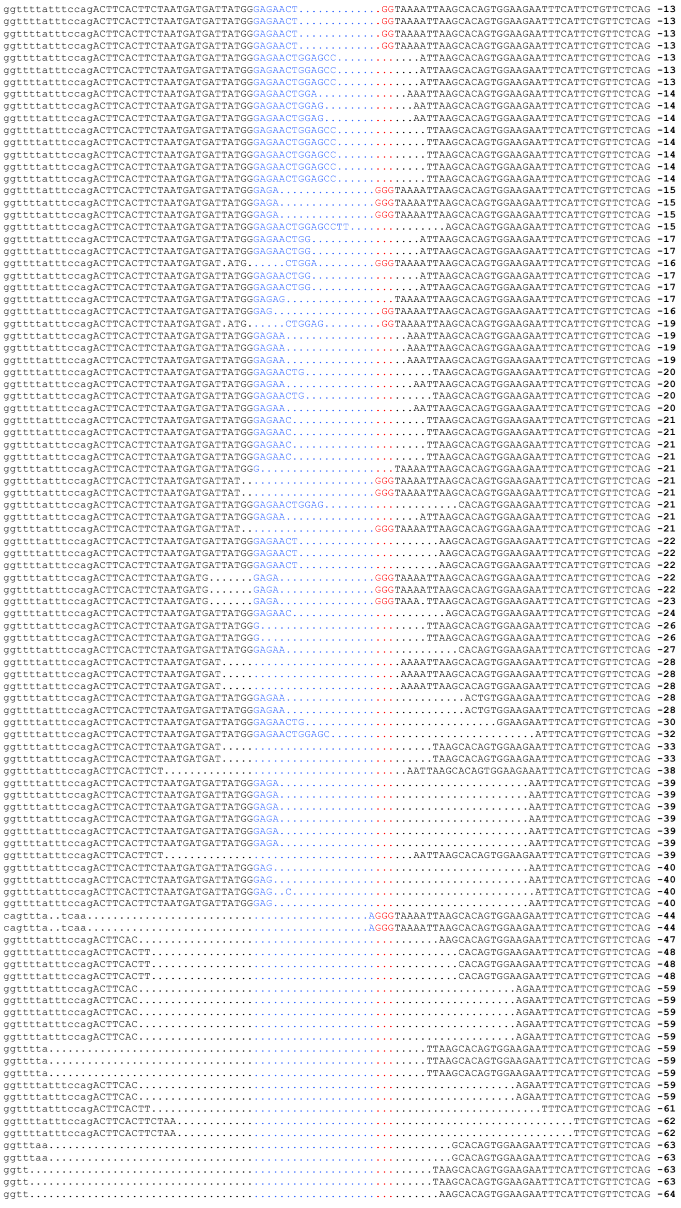

b) List of gRNA-ex11 insertions

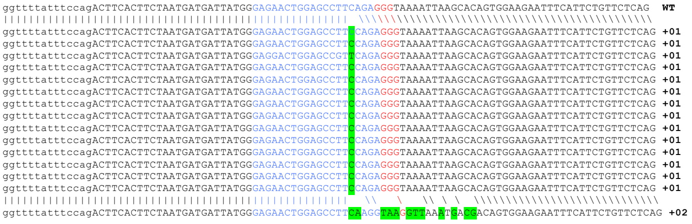

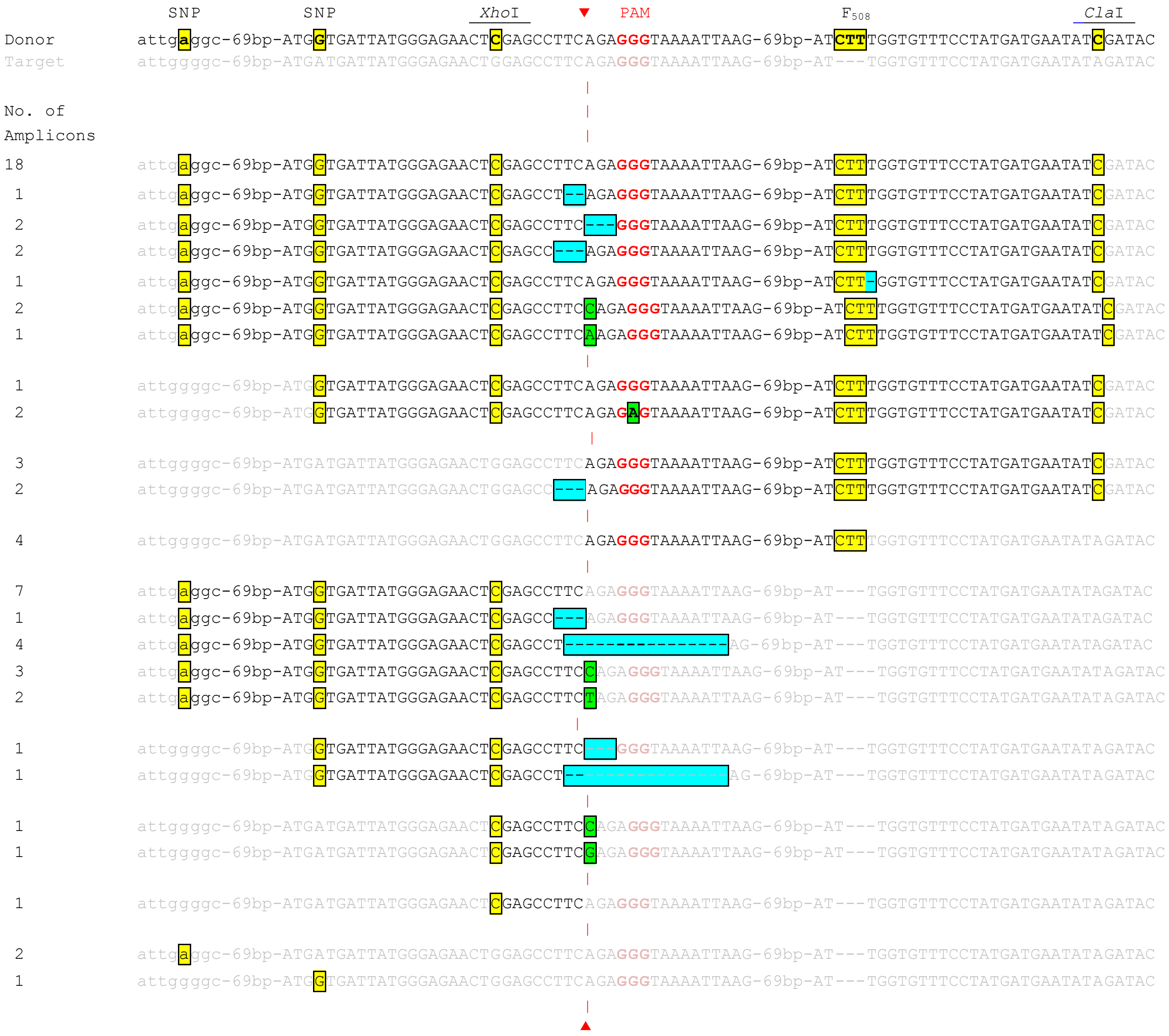

Figure S3. Sequence alignments of selected amplicons from Cas9/gRNA-ex11/donor-treated cells arranged in same order as Figure 2. Residues boxed in yellow are sequence differences in donor which are incorporated into the genome by HDR. Residues boxed in green and blue are insertions and deletions due to NHEJ repair respectively. Two 69bp regions, one either side of the gRNA target site, are identical to target sequence in all amplicons and omitted for clarity.
